# Delivery of tobacco control programs by Aboriginal Community Controlled Health Organisations in New South Wales, Australia: A cross‐sectional survey

**DOI:** 10.1002/hpja.725

**Published:** 2023-04-17

**Authors:** Jamie Bryant, Natasha Noble, Sandra Eades, Rob Sanson‐Fisher

**Affiliations:** ^1^ Health Behaviour Research Collaborative University of Newcastle Callaghan NSW Australia; ^2^ College of Health, Medicine and Wellbeing University of Newcastle Callaghan NSW Australia; ^3^ Hunter Medical Research Institute New Lambton Heights NSW Australia; ^4^ Curtin Medical School Curtin University Bentley WA Australia; ^5^ School of Population and Global Health University of Melbourne Carlton Vic. Australia

**Keywords:** Aboriginal Australians, cross sectional survey, Indigenous health services, smoking, smoking cessation

## Abstract

**Issue Addressed:**

To describe the characteristics of tobacco control programs (TCPs) delivered by Aboriginal Community Controlled Health Services (ACCHSs) in New South Wales (NSW), Australia.

**Methods:**

A key informant from each ACCHS in NSW completed a 30‐item online survey. For each TCP, ACCHSs were asked to provide: the target population group, program aims and activities, funding source, and whether the program had been monitored or evaluated and reflected principles of community control and engagement.

**Results:**

Twenty‐five of 38 eligible ACCHSs completed the survey (66% response rate). Overall, 64% of services reported currently delivering at least one TCP, almost all of which aimed to promote quitting (95%). Programs involved brief intervention for tobacco cessation (71%), referral to quit services (67%), or use of printed resources (67%). Funding sources included Local Health Districts (52% of programs), the Commonwealth Government (48%) and NSW Ministry of Health (43%). Most programs were aimed at all Aboriginal people who smoke (76%); 19% targeted women or families during pregnancy/birth. Many TCPs used culturally tailored resources (86%) and employed Aboriginal staff (86%), and 48% had been evaluated.

**Conclusions:**

A third of participating ACCHS did not have a specific TCP to address smoking among Aboriginal people, and delivery of programs was characterised by an uncoordinated approach across the state. Aboriginal staff and culturally tailored messages were a focus of existing TCP programs.

**So What?:**

Findings highlight the need for more investment in TCPs for Aboriginal people to ensure all ACCHSs can deliver evidence‐based programs.

## 1 INTRODUCTION

Despite recent declines in rates of smoking, tobacco remains the most preventable cause of illness and early death among Aboriginal people, responsible for around one in five deaths, and one‐third of cancer and cardiovascular disease.^1^ In 2018–2019 in New South Wales (NSW), 26.4% of Aboriginal people over 15 years reported smoking, which was double the rate for non‐Aboriginal people.^2^ Smoking is influenced by many social and environmental factors, and for Aboriginal people was particularly influenced by colonisation, including the introduction and use of tobacco in government rations and as payment for labour.^3^, ^4^ The ongoing impacts of colonisation and racism, and the resulting economic and social exclusion, continue to be drivers of tobacco use.^4^ The Tobacco Industry also perpetuates social and structural pressures to use tobacco through the promotion and supply of tobacco products.^4^, ^5^

Effective tobacco control to reduce smoking among Aboriginal people is essential to achieving the national priority of “closing the gap” in life expectancy between Aboriginal and non‐Aboriginal Australians, given that tobacco use is the leading contributor to the burden of disease for Aboriginal and Torres Strait Islander peoples.^6^ Aboriginal Community Controlled Health Services (ACCHSs) provide culturally appropriate primary health care services^7^ to approximately 50% of Aboriginal people nationally,^8^ and thus play a key role in providing smoking cessation support to their clients. Over two decades ago, a lack of specific tobacco control programs (TCPs) for Aboriginal and Torres Strait Islander Australians was identified.^9^ In 2010, Commonwealth action to address Aboriginal smoking was delivered through the multi‐component Tackling Indigenous Smoking and Healthy Lifestyle (TIS&HL) program, which provided funds for a dedicated workforce of healthy lifestyles workers to reduce smoking rates.^10^ The program was revised and replaced in 2015 with the Tackling Indigenous Smoking (TIS) program, which includes grant funding for regional tobacco control activities, a range of national supports for implementation, performance monitoring and evaluation and enhanced Quitlines.^10^ The TIS program was evaluated in 2018, with results suggesting it would contribute to a reduction in smoking rates.^3^, ^11^ Despite this, there is still little information available about the types of TCPs currently being delivered by ACCHSs in NSW. It remains critical to understand the types of TCPs that are currently delivered, how these programs align with available research evidence, and whether there are gaps in program delivery.

Aims: To explore among ACCHSs in NSW:

1. The number, aims and types of TCPs being delivered, including program target populations;
2. The cultural appropriateness of programs; and
3. Whether programs are regularly monitored or have been evaluated for effectiveness.

## 2 METHODS

### 2.1 Design

An online cross‐sectional survey of ACCHSs within NSW was conducted between November 2018 and January 2019.

### 2.2 Recruitment and data collection

ACCHSs in NSW were identified from the National Aboriginal Community Controlled Health Organisation website (https://www.naccho.org.au/). Services were contacted by telephone to confirm eligibility (ie, to confirm organisations were providing primary care medical services) and obtain the name and email contact for the Chief Executive Officer (CEO). The CEO was invited to participate in the study via an email from the Chief Investigator (SE), which described the study and provided a link to the Study Information Statement and online survey (administered in REDcap™). Organisations were asked to identify an individual from the organisation with good knowledge of the organisation's TCPs to complete the survey. Two follow‐up phone calls were made after 2 and 4 weeks if the survey had not been completed. A reminder email (including resending the link to the Study Information Statement and online survey) was sent to organisations where the potential respondent could not be contacted by phone, and if requested by the organisation. The survey included 30 items and took between 20 and 40 minutes to complete. Respondents were able to save their responses and complete the survey later if desired. Informed consent was implied by completion of the online survey.

### 2.3 Measures

The survey was developed for the study based on a critical review of the literature and input from an expert reference group, including several Aboriginal advisors. The online survey collected information about whether the organisation was currently providing any TCPs targeted at Aboriginal people. TCPs were defined as “any activities, services or programs provided to prevent people from taking up smoking, or assist smokers to quit.” Services who indicated they were currently providing one or more TCP/s targeted at Aboriginal people were asked to provide, for each program: the target population group (eg, all Aboriginal people, men only, women only etc.), program aims (eg, to promote quitting, to prevent uptake of smoking etc.) and activities (eg, social marketing, brief intervention, face to face counselling etc.), program name and source of funding, and whether the program had been monitored for effectiveness, or had been evaluated (this could include in terms of program usage, acceptability or outcomes). A series of questions was developed for the survey to assess whether the program reflected principles of Aboriginal control and engagement, based on the “Comprehensive approach to Aboriginal and Torres Strait Islander tobacco control” (CATs) Framework developed by the Chief Investigator (SE) and colleagues.^12^ The CATs Framework draws on the vision, principles and priorities of the National Aboriginal Torres Strait Islander Health Plan 2013–2023,^13^ and survey questions covered principles including partnership, engagement and accountability.^12^ The specific elements of Aboriginal community control and engagement measured are shown in Table 1.

**TABLE 1:** Characteristics of tobacco control programs currently being delivered by ACCHSs in NSW

Characteristics of tobacco control programs	N (% of programs^a^)
Aims of TCPs (n = 21)	
Promote quitting	20 (95%)
Reduce exposure to second‐hand smoke	13 (62%)
Promote or raise awareness of smoke free environments	12 (57%)
Increase capacity of organisations in tobacco control	8 (38%)
Prevent the uptake of smoking	10 (48%)
Reduce smoking in the workplace	6 (29%)
Promote smoke free health services	7 (33%)
Research	4 (19%)
Reduce tobacco sales and availability	2 (10%)
Other	2 (10%)
Types of TCPs (n = 21)	
Brief intervention	15 (71%)
Referral to quit smoking services	14 (67%)
Printed resources	14 (67%)
Face‐to‐face counselling and support	12 (57%)
Free or subsidised NRT	12 (57%)
Staff education and training	7 (33%)
Information sessions	7 (33%)
Telephone counselling and support	6 (29%)
Training/support of community organisation policy or projects	5 (24%)
Signage/billboards	4 (19%)
Media promotion	4 (19%)
Research and evaluation	3 (14%)
Online counselling and support	2 (10%)
Social marketing (TV, print, radio, online, cinema, social media)	5 (10%)
Policy development	1 (5%)
Funding of TCPs (n = 21)	
Local Health District	11 (52%)
Commonwealth Government	10 (48%)
NSW Ministry of Health	9 (43%)
ACCHS/Self‐funded	6 (29%)
Non‐government Organisation	3 (14%)
Other (eg, University)	4 (19%)
Target population of TCPs (n = 21)	
All Aboriginal smokers	16 (76%)
Men only	‐
Women only	‐
Pregnancy/birth	4 (19%)
Staff	‐
School students	‐
Adolescents 15 y or under	1 (5%)
Older people 65 y and over	‐
Cultural engagement in TCPs (n = 21)	
Aboriginal people/groups involved in program design	11 (52%)
Aboriginal people/groups involved in program delivery	11 (52%)
Program uses culturally‐tailored resources or messages	18 (86%)
Program includes cultural competence training for staff	11 (52%)
Program employs Indigenous staff	18 (86%)
Program monitoring and evaluation (n = 21)	
Regular monitoring of the effectiveness of the program	14 (67%)
Program has been evaluated	10 (48%)
Type of evaluation of TCPs (n = 10)	
Program usage	10 (100%)
Acceptability to users	5 (50%)
Program outcomes	5 (50%)
Program implementation	4 (40%)
Acceptability to staff	3 (30%)
Other	1 (10%)

Abbreviations: ACCHSs, Aboriginal Community Controlled Health Services; NSW, New South Wales; TCP, tobacco control program.

^a^ % May not add to 100% where respondents could select multiple responses.

### 2.4 Governance, ethics and positioning of the research team

This study was part of a larger project titled “Comprehensive approach to Aboriginal and Torres Strait Islander tobacco control,” which arose from community‐identified priorities to understand the extent to which TCPs in NSW align with principles from the National Aboriginal and Torres Strait Islander Health Plan 2013–2023,^13^ and priority areas from the National Tobacco Strategy.^14^

Aboriginal community input and oversight of the study was achieved through the leadership of the project Chief Investigator (SE), and the support of the CEO of an Aboriginal Medical Service in NSW, who ensured the research privileged Aboriginal and Torres Strait Islander world views. The research team contributed Aboriginal lived experience (SE), as well as specific experience in public health and tobacco control research (all authors). At all times, the research team was committed to conducting meaningful research with accountability to Aboriginal and Torres Strait Islander communities.

This research was carried out with reference to the principles contained in Ethical conduct in research with Aboriginal and Torres Strait Islander Peoples and communities: Guidelines for researchers and stakeholders 2018^15^ and Keeping research on track II 2018.^16^ Ethical approval for the study was granted by NSW Aboriginal Health and Medical Research Council Human Research Ethics Committee (AHMRC HREC; Ref: 1436/18), Hunter New England HREC (Ref: 18/08/15/4.05) and University of Newcastle HREC (Ref: H‐2018‐0411). Approval for publication of the manuscript was also obtained from the NSW AHMRC HREC.

## 3 RESULTS

Twenty‐five of the 38 eligible ACCHSs from NSW completed the survey (representing a 66% response rate). Using 2016 Australian Statistical Geography Standard‐Remoteness Areas, ACCHSs who completed the survey were located in major cities (n = 3; 12%), inner regional (n = 11; 44%), outer regional (n = 7; 28%) and remote or very remote areas of NSW (n = 4; 16%). This was very similar to the distribution of all ACCHSs in NSW (13%, 45%, 26%, 16%, respectively).

All survey results are presented in Table 1.

### 3.1 Number and aims of TCPs being delivered

Sixteen of the 25 services (64%) were currently delivering one or more TCPs. Eight services were not currently delivering a TCP; and one service did not provide a program but provided quit smoking resources (such as brochures, website information). Five services (20%) were delivering more than one TCP. As a result, data were provided on a total of 21 different TCPs. Most TCPs aimed to promote quitting (95%). A majority also aimed to reduce exposure to second‐hand smoke (62%), and/or to promote or raise awareness of smoke free environments (57%).

### 3.2 Types and target populations of TCPs

Most programs involved brief intervention for tobacco cessation (71%), referral to quit smoking services (67%), use of printed quit resources (67%), face to face counselling and support (57%) and provision of free or subsidised nicotine replacement therapy (NRT; 57%). The programs being delivered included the TIS program (n = 6; 29% of programs), Quit for New Life (n = 3; 14%) and SISTAQUIT (n = 2; 10%). Other programs included “Smoke free for life,” “Beat the Boondah” and “Yarning about Quitting,” or were described more generally as “smoking cessation” or “Aboriginal tobacco control projects.” Most programs were aimed at all Aboriginal people who smoke (76%). Four programs (19%) were focused on pregnancy and birth, and targeted pregnant women, women who had recently given birth, and/or Aboriginal parents, partners, and families of Aboriginal babies.

### 3.3 Funding of TCPs

The current programs were receiving funding from a range of sources, most frequently Local Health Districts (52% of programs), the Commonwealth Government (48% of programs) and the NSW Ministry of Health (43% of programs). Almost one in three of the programs (29%) were receiving funding from multiple funding sources.

### 3.4 Community control and engagement in TCPs

Almost all TCPs used culturally tailored resources or messages (86%) and employed Aboriginal staff to deliver the programs (86%). Over half of the programs had involved Aboriginal people or groups in their design and delivery (52%) and included cultural competence training for staff delivering the program (52%).

### 3.5 Monitoring and evaluation of TCPs

Approximately two‐thirds of the programs were regularly monitored for their effectiveness (67%), while less than half (48%) had been evaluated. Of the evaluated programs (n = 10), all had been assessed for program usage (ie, number of people who had accessed or been reached by the program; 100%); half had been assessed in terms of acceptability to participants (n = 5, 50%), and half had been evaluated for program outcomes (ie, number of people who quit or quit attempts, or other changes in behaviour; n = 5, 50%).

## 4 DISCUSSION

Almost two‐thirds of participating ACCHSs in NSW reported currently providing a TCP, which in the current study could include the provision of free or subsidised NRT. One‐third of ACCHSs reported they did not have a specific program aimed at preventing or reducing smoking among Aboriginal people. Previous national research conducted in 2015 reported similar findings, with just over half of surveyed services (17/32; 53%) running programs to help people quit smoking (including Aboriginal specific tobacco control and healthy lifestyle programs, and mainstream quit programs), and 78% of services (25/32) having prescribed or dispensed free NRT.^17^ The apparent gap in the provision of TCPs by NSW ACCHSs identified in the current study is of concern, given continuing relatively high rates of smoking among Aboriginal people and the significant harms associated with smoking.^2^

In the current study, there were a range of different TCPs being implemented. The most commonly reported was the TIS program, which aims to reduce smoking through population health promotion activities. Quit for New Life (an LHD based program promoting quitting during pregnancy and after birth)^18^ and SistaQuit (a trial aiming to improve healthcare provider skills in smoking cessation care for pregnant Aboriginal women)^19^ were being delivered in three and two services, respectively. Such programs were only identified as being implemented in a minority of ACCHSs across the state, with many services delivering other or unnamed programs, which may or may not be evidence based. Programs were most often being funded by Local Health Districts, Commonwealth, and/or State funding (and sometimes a combination of these), with almost a third of TCPs being funded by the ACCHS themselves (presumably from untied funds). The range of programs and program funding in the current study highlights a somewhat fragmented approach to program delivery and funding within the state of NSW, and supports calls for coordination and dedicated funds for the delivery of locally tailored Aboriginal TCPs.^9^ For example, the 2010 TIS&HL program funded a national workforce of tobacco action workers and coordinators across 57 regions.^20^ The 2015 revised TIS program included regional grants, and in 2018, regional tobacco control grants were being provided to 37 organisations nationally,^10^, ^20^, ^21^ the majority of which were ACCHSs.^3^ It is estimated that nationally, more than a quarter of Aboriginal and Torres Strait Islander peoples live outside of TIS serviced regions.^22^ Despite additional funding committed to the TIS by the Federal Government in 2022,^23^ a significant proportion of ACCHSs nationally are not receiving specific TCP funding through the TIS.

Most of the TCPs in the current study were aimed at all Aboriginal people who smoke, with a proportion of programs targeting women who were pregnant or had recently given birth (including partners and families of new babies). The focus on pregnancy and birth aligns with disproportionately high levels of smoking among Aboriginal women during pregnancy,^24^ and well documented adverse impacts such as a higher risk of still birth, preterm deliveries and low birthweights.^25^, ^26^ However, there may be scope for additional programs which are targeted and tailored towards other age groups, for example, the prevention of smoking uptake and cessation for Aboriginal adolescents.^11^

Most current TCPs aimed to promote quitting and reduce exposure to second‐hand smoke. Given the high prevalence of current smoking, the focus on quitting is appropriate. However, additional or expanded TCPs could aim to prevent the initiation/uptake of smoking, and target workplace smoking or smoke‐free health services. The latter is important given many ACCHS health workers also smoke.^11^

The most common TCP activities were brief intervention for cessation, referral to quit smoking services, face to face counselling, free or subsidised NRT and provision of printed resources. This is consistent with the aim of most current TCPs to promote quitting, and reflects best‐practice evidence for the effectiveness of brief intervention and pharmacological approaches for smoking cessation.^12^ There was less focus on social marketing/social media initiatives, or offering online or telephone support for cessation, although these are more likely to be implemented by larger State or National organisations. Approaches which utilise social media and offer alternative access to support may help to broaden the reach of current TCPs, particularly for younger Aboriginal people.

There was good alignment of current TCPs with the principles of community control and engagement as assessed in the survey. Almost all programs reported the use of culturally tailored resources or messages and employing Aboriginal staff for program delivery. There was also evidence of engagement of Aboriginal people in the co‐design and delivery of half of the current programs, which is important to ensure that TCPs are culturally sensitive and appropriate. Regular monitoring of the majority of current programs was also encouraging. However, less than half of the current TCPs had been evaluated for effectiveness. This is similar to the 2015 national research cited above, where less than a third of TCPs had been evaluated.^17^ In addition, program usage (ie, the number of people who had accessed or been reached by the program) was more likely to be evaluated than outcomes such as quit attempts or abstinence rates. The lack of evaluation is not surprising given that many TCPs, and other health programs more broadly, are often not evaluated.^27^ For example, the 2018 evaluation of the TIS program suggested it is meeting its short‐term goals (including growth in partnerships and community engagement, increased access to culturally appropriate quitting support, and an increase in the use of evidence to inform program design), but the long term impact in relation to a reduction of smoking rates was said to be outside the scope of the evaluation.^10^ A more recent national 2018–2020 study used a cohort design to examine smoking‐related attitudes and behaviours among Aboriginal and Torres Strait Islander adults.^3^ The Mayi Kuwayu Study found no differences in overall smoking prevalence, but some significant differences in smoking behaviours (such as a lower prevalence of smoking inside the home) among respondents living in TIS funded areas, compared to non‐TIS funded areas.^3^ Australia has implemented a range of national population level initiatives to help reducing smoking, such as investment in national social marketing, graphic health warnings on packaging, increased smoke‐free legislation, the introduction of tobacco plain packaging from 2012 and annual excise increases from 2013.^11^ In 2021, new Medicare Benefits Scheme items were introduced for patients to access nicotine and smoking cessation counselling through General and Other Medical Practitioners and Other Medical Practitioners.^28^ While these national efforts are likely to have contributed to overall declining smoking rates among Aboriginal and Torres Strait Islander Australians, regional variations remain, and reinforce the need for tailored and targeted TCPs to meet local needs.^22^, ^29^ Rigorous evaluation of these policies and programs are needed to ensure that limited funding and resources are being used effectively.

## 5 LIMITATIONS

The 66% response rate means not all TCPs being delivered by NSW ACCHS are represented in the findings, although services who did not respond may not have been delivering any TCPs. The ACCHS staff member completing the survey may not have been aware of all the services or programs delivered by their service, and some of the details of programs being implemented (such as the name of the program or the funding source). Conversely, survey respondents may have overstated what was being provided. There is also likely to be unfunded tobacco control work being undertaken by ACCHSs across NSW which was not captured in the current survey. The measures used to assess alignment of TCPs with principles of Aboriginal community control and engagement were limited, given space constraints and the quantitative nature of the survey. Finally, only data on current TCPs were presented, meaning information about past programs or future planned programs was not included.

## 6 CONCLUSION

ACCHSs in NSW are delivering a range of TCPs, which generally promote quitting and target all Aboriginal people who smoke, with a proportion also aimed at Aboriginal women and families during pregnancy and birth. However, a significant proportion of services were not currently providing a TCP, and the range of programs and funding was suggestive of a somewhat fragmented and uncoordinated approach across the state. Despite recent declines in smoking rates among Aboriginal people,^1^ and the implementation of the national TIS program, the need for a more investment in TCPs for Aboriginal people is still apparent to ensure all ACCHSs can deliver evidence based TCPs and continue to reduce the gap in smoking rates between Aboriginal and non‐Aboriginal Australians. The scope of programs being implemented could also be expanded to support healthcare staff to quit; prevent initiation of smoking; target young Aboriginal people; offer alternative engagement with programs (eg, telephone or online); and ensure delivered programs are evaluated for effectiveness in achieving smoking cessation. ACCHSs are highly committed and well placed to help their communities address smoking.^11^, ^17^, ^30^ Services should be further supported to provide and evaluate comprehensive TCPs for Aboriginal people in NSW.

## FUNDING INFORMATION

This work was funded by a grant received from the Australian Partnership Prevention Centre.

## CONFLICT OF INTEREST STATEMENT

The authors declare no conflict of interest.

## ETHICS STATEMENT

This study was approved by the Aboriginal Health and Medical Research Council Ethics Committee (1436/18) and the University of Newcastle Human Research Ethics Committee (H‐2018‐0411).

## Data Availability

The data that support the findings of this study are available from the corresponding author upon reasonable request.
